# Umbilical Cord-Derived Mesenchymal Stem Cells Inhibit Cadherin-11 Expression by Fibroblast-Like Synoviocytes in Rheumatoid Arthritis

**DOI:** 10.1155/2015/137695

**Published:** 2015-05-18

**Authors:** Cheng Zhao, Lu Zhang, Wei Kong, Jun Liang, Xinyun Xu, Hongyan Wu, Xuebing Feng, Bingzhu Hua, Hong Wang, Lingyun Sun

**Affiliations:** ^1^Department of Rheumatology and Immunology, Drum Tower Clinical Medical College of Nanjing Medical University, Nanjing, Jiangsu 210008, China; ^2^Department of Pathology, Drum Tower Clinical Medical College of Nanjing Medical University, Nanjing, Jiangsu 210008, China

## Abstract

This study aimed to determine whether umbilical cord-derived mesenchymal stem cells (UCMSC) regulate Cadherin-11 (CDH11) expression by fibroblast-like synoviocytes (FLS) in rheumatoid arthritis (RA). FLS were isolated from the synovium of RA and osteoarthritis (OA) patients. FLS from RA patients were cocultured with UCMSC in a transwell system. CDH11 mRNA levels in FLS were tested, and levels of soluble factors expressed by UCMSC, such as indoleamine 2,3-dioxygenase (IDO), hepatocyte growth factor (HGF), and interleukin- (IL-) 10, were determined. IDO, HGF, and IL-10 were upregulated in cocultures, so that appropriate inhibitors were added before determination of CDH11 expression. The effects of UCMSC on arthritis were investigated in the collagen-induced arthritis (CIA) model in Wistar rats. FLS from RA patients expressed higher CDH11 levels than those from OA patients, and this effect was suppressed by UCMSC. The inhibitory effect of UCMSC on CDH11 expression by FLS was abolished by suppression of IL-10 activity. CDH11 expression in synovial tissues was higher in the context of CIA than under basal conditions, and this effect was prevented by UCMSC administration. IL-10 mediates the inhibitory effect of UCMSC on CDH11 expression by FLS, and this mechanism might be targeted to ameliorate arthritis.

## 1. Introduction

Rheumatoid arthritis (RA) is a chronic inflammatory disease characterized by progressive destruction of joint. The primary site of inflammation in RA is the synovium, and hyperplasia of the synovial intimal lining is a hallmark of this disease. The synovial intimal lining is a loosely organized collection of cells that forms an interface between the synovium and the synovial fluid space. Macrophage-like cells and fibroblast-like synoviocytes (FLS) are the two major cell types in the lining. The intimal lining cells lack tight junctions and a definite basement membrane. Cadherins are single-pass transmembrane glycoproteins that mediate homophilic adhesion between cells [1]. Cadherin-11 (CDH11) is a type II cadherin predominantly expressed by FLS but not by macrophages or other cells of hematopoietic origin residing in the synovium. CDH11 plays a prominent role in the formation and organization of the synovial lining layer [2]. Recent research showed that CDH11 could regulate inflammation mediated by FLS [3] and promote migration of FLS and erosion of cartilages and bones [4]. This evidence indicates that CDH11 expressed by FLS plays an important role in RA pathogenesis.

Umbilical cord-derived mesenchymal stem cells (UCMSC) are multipotent stem cells that exhibit immune regulatory functions. UCMSC were reported to decrease the levels of proinflammatory cytokines and inhibit joint swelling and cartilage erosion. In the past, our team has carried out UCMSC transplants in RA patients, a treatment that improved symptoms of the disease [5]. However, the mechanisms that mediated the beneficial effects of UCMSC in RA patients, such as prevention of cartilage erosion, remain unclear.

In this study, we explored the effects of UCMSC transplantation on the expression of CDH11 in FLS from RA patients, and we investigated the mechanism whereby UCMSC ameliorate RA symptoms.

## 2. Materials and Methods

### 2.1. Harvesting of the Synovium and Umbilical Cord

Synovium samples were obtained from thirteen patients undergoing total knee arthroplasty at Drum Tower Clinical Medical College of Nanjing Medical University. Eight patients fulfilled the American College of Rheumatology criteria for the classification of RA and they had no other autoimmune or systemic diseases. One of the patients was male and seven were females, with the average age of 56.1 ± 11.1 years. Their average disease duration was 10.5 ± 5.8 years. Synovial tissues were also obtained from five osteoarthritis (OA) patients, 2 males and 3 females, with the average age of 56.8 ± 7.2 years. Their average disease duration was 8.0 ± 3.4 years. Umbilical cords were resected under sterile conditions during two natural deliveries in Drum Tower Clinical Medical College of Nanjing Medical University. The study protocol was approved by the ethics committee of the Drum Tower Clinical Medical College of Nanjing Medical University. Written informed consent was obtained from all donors.

### 2.2. Isolation and Culture of FLS and UCMSC

Synovial tissues were obtained from RA and OA patients and minced under sterile conditions. Synovial tissues were digested with collagenase I (Sigma-Aldrich, Saint Louis, Missouri, USA) at a concentration of 1 mg/mL for 4 hours (37°C, 5% CO_2_), collected, and washed twice with phosphate buffered saline (PBS). Subsequently, cells were obtained by centrifugation and cultured in DMEM/F12 with 10% fetal bovine serum (FBS) (Gibco, Australia), which was changed every three days. Upon reaching 80% confluence, cells were detached from the culture substrate by exposure to 0.25% Trypsin-EDTA (Gibco, USA) and seeded on a surface 3 times larger than the original culture substrate. After 3 passages, expression of FLS markers was documented, and cells were used in the described studies.

Wharton jelly was obtained from umbilical cords following removal of the vessels and subsequently minced. Fragments were transferred to a culture flask in the presence of DMEM/F12 with 10% FBS. Every 7 days, half of the culture medium was changed. Adherent cells at the bottom of the culture flask were digested by 0.25% Trypsin-EDTA and passaged. Subsequently, the culture medium was changed every 3 days. After 3 passages, flow cytometry was carried out to identify UCMSC with selected mesenchymal stem cells surface markers, such as CD14, CD29, CD34, CD44, CD45, CD73, CD90, and HLA-G (eBioscience, USA). Cells from passages 3 to 8 were used in the reported studies.

### 2.3. Flow Cytometry

After 3 passages in culture, FLS and UCMSC were detached from the culture substrate and washed in PBS. FLS and UCMSC were incubated with appropriate antibodies, such as fluorescein isothiocyanate- (FITC-) conjugated anti-HLA-G, CD4, CD14, CD29, CD34, CD44, CD45, CD55, CD73, and CD90 (eBioscience, USA) for surface marker staining and then were maintained in the dark at 4°C for 30 min before washing and resuspension in PBS. These surface markers were detected by flow cytometry.

### 2.4. Coculture of FLS and UCMSC

UCMSC were seeded on transwells with a 0.4 *μ*m pore size, whereas FLS from RA patients were seeded on 6-well plates. After reaching confluence, FLS were transferred to the transwell system and cocultured with UCMSC for 72 hours. Subsequently, cells were harvested to measure CDH11 expression in FLS and levels of soluble factors produced by UCMSC. Selected soluble factors inhibitors such as 1-MT, or an anti-IL-10 or anti-HGF antibodies (R&D Systems, Minneapolis, USA) were added to the coculture system.

### 2.5. Collagen-Induced Arthritis (CIA) and Cell Transplantation

Wistar rats were purchased from Vital River Laboratory Animal Technology Co. Ltd. The CIA procedure entailed emulsification of collagen type II (CII) with Freund's complete adjuvant (Sigma-Aldrich, Saint Louis, Missouri, USA) and administration of the emulsion to 8-week-old rats by intracutaneous injection. After 14 days, rats were administered a second injection of CII emulsified with Freund's incomplete adjuvant. Based on clinical scores, rats were monitored for signs of arthritis onset. Clinical arthritis was scored on a scale of 0 to 3, where 0 = no swelling, 1 = slight swelling and erythema, 2 = pronounced edema, and 3 = joint rigidity. Each limb was graded, and the grades were summed to yield the arthritis score for each animal (maximum possible score 12 per animal) [6]. After 17 days, 1 × 10^6^ UCMSC or FLS or 1 mL PBS was administered by tail vein injection [7]. Rats were sacrificed after 42 days, and synovial tissues were collected.

### 2.6. Histologic Analysis

Formalin-fixed limbs were decalcified and paraffin-embedded using standard histologic techniques. Serial 4 *μ*m sections were cut and stained with hematoxylin and eosin to examine morphologic features.

Formalin-fixed, paraffin-embedded tissue sections of 3 *μ*m were placed on adhesive-coated slides. In the process of a heated antigen retrieval process, the slides were immersed in EDTA buffer (pH 8.0) and heated for 2 min in the steamer. The slides were incubated overnight at 4°C with monoclonal antibodies to CDH11 (P707, Abcam) with 1 : 50 dilution in bovine serum albumin before incubating with Envision Detection System (k5007, Dakocytomation, Glostrup, Denmark) at room temperature for 20 min. Color was developed by reaction with DAB solution for 10 min followed by counterstaining with Harris hematoxylin, dehydrated, coverslipped, and reviewed under light microscope.

### 2.7. RNA Isolation and Real-Time PCR

Cocultured cells, FLS from OA patients, and synovium from rats subjected to CIA were harvested in 500 *μ*L of Trizol (Invitrogen, Van Allen Way, Carlsbad, California); 70 *μ*L chloroform was added, and the solution was mixed. Subsequently, the sample and Trizol/chloroform mix were centrifuged at 11440 r/min for 15 min, the upper layer was collected, and an equal volume of isopropanol was added before incubation at room temperature for 10 min. Samples were centrifuged at 11440 r/min for 15 min and the supernatant was discarded. The total RNA pellets were washed with 75% ethanol and dissolved with water treated with diethyl pyrocarbonate (DEPC) 0.05 *μ*g/*μ*L. Reverse transcription of mRNA was carried out with Reverse transcription kits.

The real-time PCR reaction contained SYBR Premix Ex Taq 10 *μ*L, ROX Reference Dye (50x) (Takara, DaLian, China) 0.4 *μ*L, cDNA 2 *μ*L, and dH_2_O 6.8 *μ*L, for a total volume of 20 *μ*L. All reactions were conducted in duplicate. The 2^−ΔΔ*Ct*^ method was used to normalize expression of target genes mRNA to glyceraldehyde 3-phosphate dehydrogenase (GAPDH) expression [8]. Primers for real-time PCR were shown in Table 1.

### 2.8. Western Blot

Cells were washed with ice-cold PBS and lysed in RIPA lysis buffer for 30 min on ice. Equal amounts of protein of cell lysates were separated on 10% SDS-polyacrylamide gel, transferred to nitrocellulose membrane, and analysed using anti-CDH11 (P707) antibody (Cell Signaling Technology). Horseradish-peroxidase-conjugated goat anti-rabbit immunoglobulin G (Jackson ImmunoResearch) was used as secondary antibody. The target proteins were detected using an enhanced chemiluminescence detection kit (Millipore).

### 2.9. Statistical Analysis

Data are expressed as means ± SD. SPSS 13.0 was used to analyze data and GraphPad Prism 5 to draw graphs. Two independent groups were compared by unpaired *t*-test and multiple independent groups by one-way analysis of variance. *P* < 0.05 was considered significant.

## 3. Results

### 3.1. Identification of UCMSC and FLS

**Figure 1:**
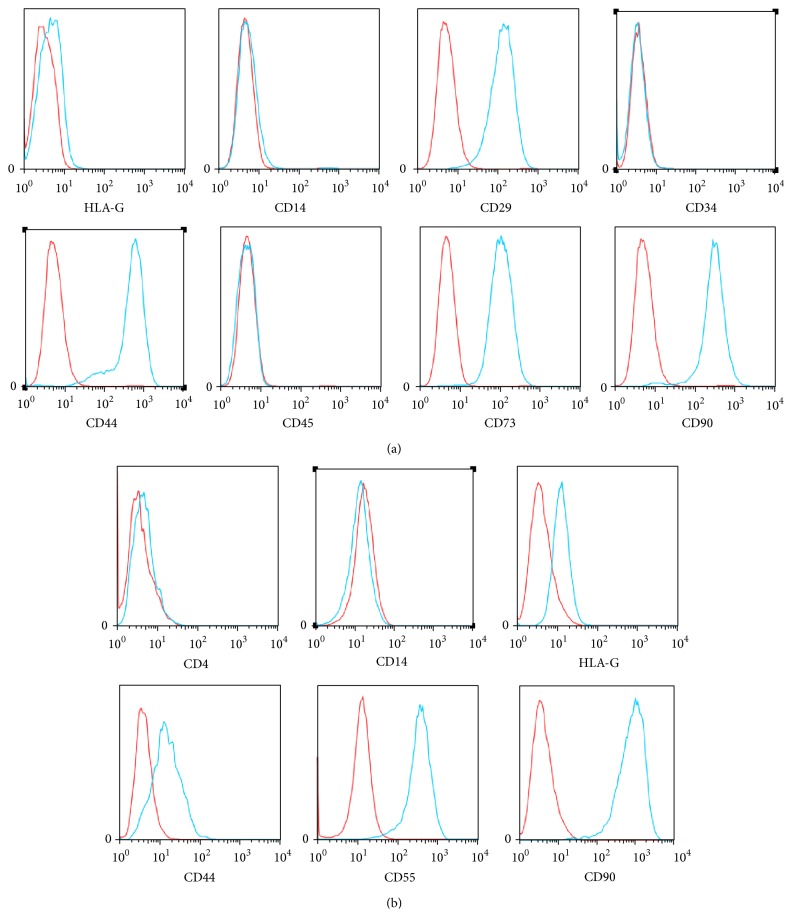
Identification of UCMSC and FLS. (a) Expression of the UCMSC markers: HLA-G, CD14, CD29, CD34, CD44, CD45, CD73, and CD90. (b) Expression of FLS markers: HLA-G, CD4, CD14, CD55, CD44, and CD90.

To ensure the MSC nature of UCMSC, we harvested umbilical cord-derived cells after 3 passages in culture and detected selected cell surface markers, such as HLA-G, CD14, CD29, CD34, CD44, CD45, CD73, and CD90 by flow cytometry. Cells isolated from umbilical cords were positive for CD29, CD44, CD73, and CD90 but negative for HLA-G, CD14, CD34, and CD45 (Figure 1(a)).

In the joints of RA patients, synoviocytes could be classified in scavenger synovial cells and FLS. Furthermore, inflammatory T cells were observed in the joint. Therefore, presence of the T cell marker CD4, of the macrophage marker CD14, and of HLA-G, CD44, CD55, and CD90 was tested in synoviocytes after 3 passages in culture. Neither CD4 nor CD14 was detected, whereas HLA-G was expressed marginally, indicating low immunogenicity. Cells were positive for the fibroblast markers CD44 and CD90. More than half of the synoviocytes expressed CD55, meaning that the majority of FLS originated from the lining layer (Figure 1(b)).

### 3.2. UCMSC Inhibit CDH11 Expression in FLS from RA Patients In Vitro

**Figure 2:**
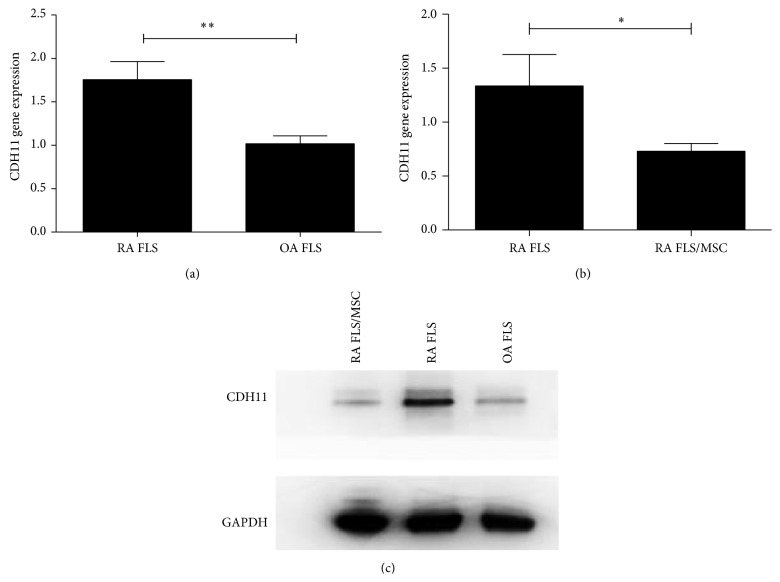
Effects of UCMSC on CDH11 expression by FLS from RA patients (a)–(c). Expression of CDH11 mRNA (a) and protein (c) by FLS from RA patients. RA: rheumatoid arthritis; OA: osteoarthritis; MSC: mesenchymal stem cells; CDH11: cadherin-11. ^∗^ *P* < 0.05; ^∗∗^ *P* < 0.01 (b)-(c). Expression of CDH11 mRNA (b) and protein (c) by FLS from RA patients, cocultured with UCMSC. RA: rheumatoid arthritis; OA: osteoarthritis; MSC: mesenchymal stem cells; CDH11: cadherin-11. ^∗^ *P* < 0.05; ^∗∗^ *P* < 0.01.

Since CDH11 is critical for cartilage destruction in RA, we compared CDH11 expression by FLS from RA and OA patients. CDH11 expression was higher in FLS from RA patients than in those from OA patients at the mRNA (1.76 ± 0.51 versus 1.02 ± 0.20, *P* < 0.01) and protein level (Figures 2(a)–2(c)).

Since we reported that UCMSC transplantation could ameliorate RA symptoms, we hypothesized that UCMSC might inhibit cartilage erosion by decreasing CDH11 expression in FLS. To test this hypothesis, we cocultured RA FLS with UCMSC and measured changes in CDH11 expression. UCMSC downregulated CDH11 expression in FLS at the mRNA (1.34 ± 1.24 versus 0.73 ± 0.30, *P* < 0.05) and protein level (Figures 2(b) and 2(c)).

### 3.3. Changes in Levels of Soluble Factors Expressed by UCMSC

**Figure 3:**
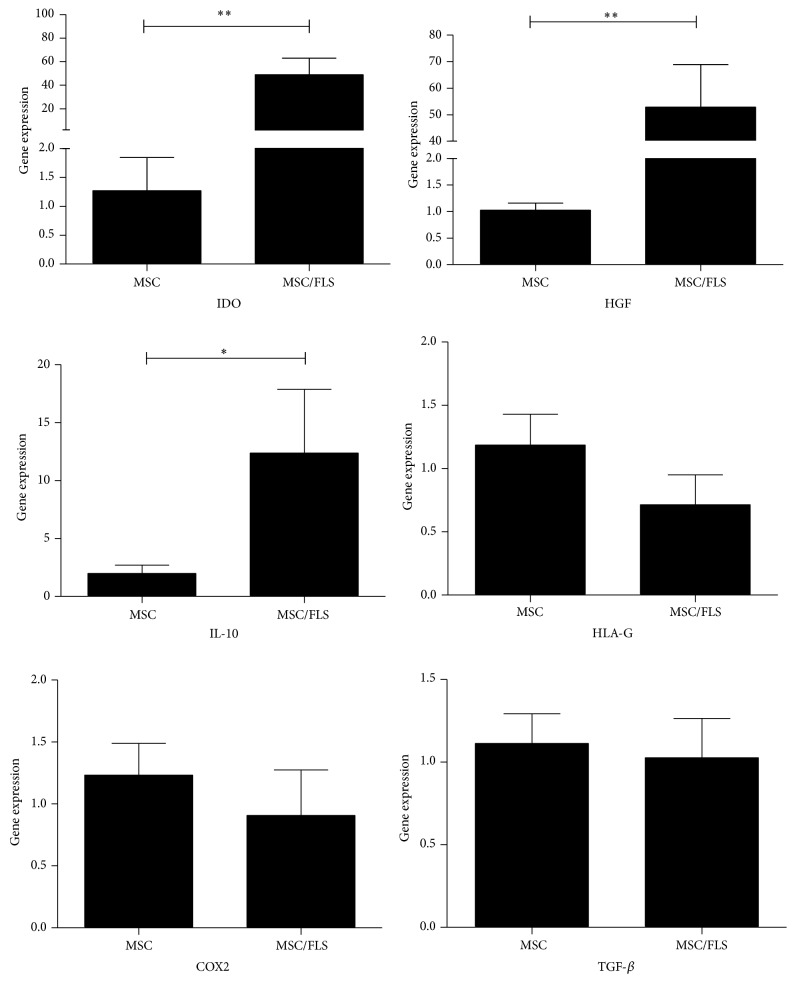
Soluble factors expressed by UCMSC. Expression of COX-2, HGF, HLA-G, IDO, TGF-*β*, and IL-10 by UCMSC cocultured with FLS. MSC: mesenchymal stem cells; FLS: fibroblast-like synoviocytes. ^∗^ *P* < 0.05; ^∗∗^ *P* < 0.01.

UCMSC and FLS cocultured in the transwell systems were not in direct contact. Therefore, soluble factors mediated the inhibitory effects of UCMSC on CDH11 expression in FLS. In previous studies, multiple factors such as IDO, cyclooxygenase2 (COX2), HGF, IL-10, transforming growth factor- (TGF-) *β*, and human leukocyte antigen- (HLA-) G were reported to facilitate the inhibitory effects of MSC on inflammation. Expression of these soluble factors by UCMSC was tested, and we observed that IDO (1.27 ± 1.00 versus 48.90 ± 44.79, *P* < 0.05), HGF (1.03 ± 0.26 versus 52.85 ± 55.69, *P* < 0.05), and IL-10 (1.99 ± 2.00 versus 12.38 ± 20.58, *P* < 0.05) were upregulated in cocultures with FLS. Conversely, changes in COX2 (1.23 ± 0.77 versus 0.91 ± 1.10, *P* > 0.05), TGF-*β* (1.11 ± 0.57 versus 1.03 ± 1.04, *P* > 0.05), or HLA-G (1.19 ± 0.73 versus 0.71 ± 0.71, *P* > 0.05) expression were modest (Figure 3).

### 3.4. Suppression of IL-10 Prevents the Inhibitory Effects of UCMSC on CDH11 Expression

**Figure 4:**
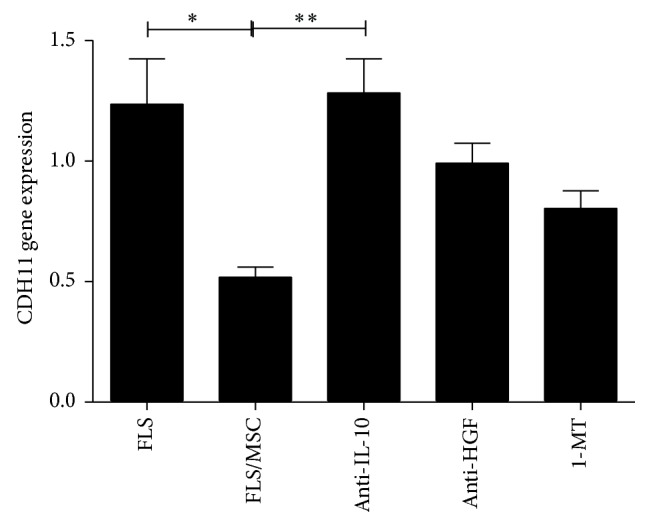
Suppression of soluble factors prevented the inhibitory effect of UCMSC on CDH11 expression by FLS from RA patients. Expression of CDH11 by FLS from RA patients, cocultured with UCMSC in the presence of anti-IL-10, anti-HGF antibody, or 1-MT. MSC: mesenchymal stem cells; FLS: fibroblast-like synoviocytes; CDH11: cadherin-11. ^∗^ *P* < 0.05; ^∗∗^ *P* < 0.01.

We reported that UCMSC cocultured with FLS from RA patients expressed higher levels of IDO, HGF, and IL-10, and these molecules might mediate the inhibitory effect of UCMSC on CDH11 expression by FLS. Therefore, UCSMC and FLS were cocultured in the presence of IDO, HGF, and IL-10 inhibitors to determine the contribution of each factor to the effect of UCMSC on CDH11 expression by FLS. An anti-IL-10 antibody opposed the inhibitory effect of UCMSC on CDH11 expression (1.28 ± 0.60 versus 0.52 ± 0.10, *P* < 0.01). Conversely, an anti-HGF antibody (1.32 ± 1.44 versus 0.52 ± 0.10) or the IDO inhibitor 1-MT (1.28 ± 1.58 versus 0.52 ± 0.10) had no impact on the effect of UCMSC on CDH11 expression (Figure 4).

### 3.5. UCMSC Transplantation Prevented Tissue Damage in CIA

**Figure 5:**
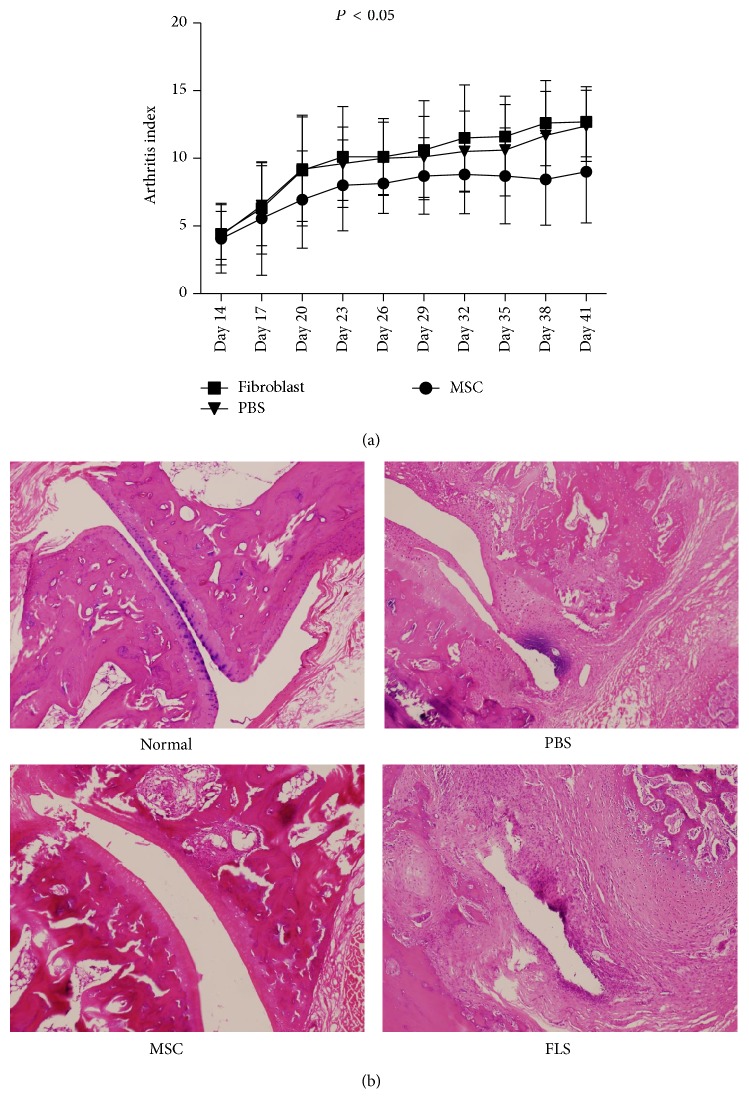
UCMSC prevented tissue damage in CIA. (a) The severity of CIA was progressively attenuated in UCMSC treated rats, as compared with PBS and FLS treated rats. *P* < 0.05. (b) H&E-stained sagittal sections of joints from CIA rats. PBS and FLS treated rats showed a marked severe synovitis and cartilage erosion. However, the majority of joints from mice injected with UCMSC had normal morphology with a smooth articulation cartilage surface. Magnification, 100x. UCMSC: umbilical cord-derived mesenchymal stem cell; FLS: fibroblast-like synoviocytes; CIA: collagen-induced arthritis.

As shown in Figure 5(a), the severity of CIA was progressively attenuated in UCMSC treated rats, as compared with FLS and PBS treated rats. The therapeutic effects of UCMSC on CIA in rats were further verified by histological examination at the endpoint of clinical study. We observed that control rats exhibited a marked severe synovitis, cartilage erosion. In contrast, the majority of joints from rats injected with UCMSC had normal morphology with a smooth articulation cartilage surface (Figure 5(b)).

### 3.6. UCMSC Transplantation Decreased CDH11 Levels in the Synovial Tissue of Rats Subjected to CIA

**Figure 6:**
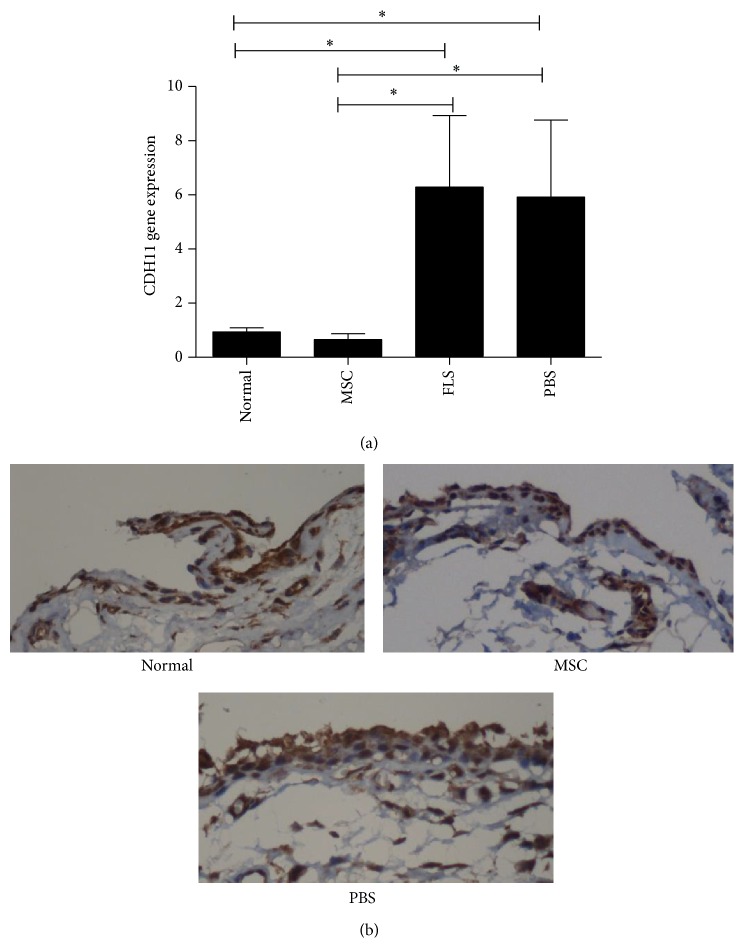
UCMSC transplantation inhibited expression of CDH11 induced by CIA in synovial tissue of rats. (a) Rats subjected to CIA were administered PBS, UCMSC, or fibroblasts. CDH11 mRNA levels in synovial tissues of rats subjected to CIA and administered UCMSC were similar to those in synovial tissues of normal rats, whereas which were lower than in the context of fibroblast or PBS administration. ^∗^ *P* < 0.05. (b) The expression of CDH11 increased remarkably in synovial tissues of rats subjected to CIA, whereas which decreased significantly after UCMSC transplantation. Magnification, 400x. UCMSC: umbilical cord-derived mesenchymal stem cell; CIA: collagen-induced arthritis; CDH11: cadherin-11.

UCMSC have negligible immunogenic potential, and as a consequence these cells have been used in therapies for autoimmune diseases in both humans and animals. UCMSC were transplanted in rats subjected to CIA, and PBS or fibroblasts were used as control. Following transplant, CDH11 mRNA levels in synovial tissues of rats subjected to CIA and administered UCMSC were similar to those in synovial tissues of normal rats (0.65 ± 0.91 versus 0.94 ± 0.46). Accordingly, CDH11 mRNA levels in rats administered UCMSC were lower than which in synovial tissues of rats subjected to CIA, either in the context of fibroblast or PBS administration (0.65 ± 0.91 versus 6.29 ± 6.45 versus 5.92 ± 7.51) (Figure 6(a)). Our immunohistochemical data showed that the expression of CDH11 increased remarkably in synovial tissues of rats subjected to CIA, whereas it decreased significantly after UCMSC transplantation (Figure 6(b)).

## 4. Discussion

RA is an autoimmune disease characterized by chronic proliferation of synovial cells and progressive joint damage. In the synovium of RA patients, proliferation of FLS, the most prominent cells in the lining layer, plays an important role in thickening of the synovium. Previous research demonstrated that activated FLS cause arthritis and cartilage degradation [9, 10]. In addition, it was reported that FLS act as cancer-like cells and are able to migrate and cause inflammation and degradation of other joints [11].

FLS express CDH11, an adhesion molecule that might mediate formation of the lining layer and preserve the integrity of the synovial tissue by mediating FLS-to-FLS interactions. A previous study demonstrated that the normal synovial lining layer was absent in* CDH11* ^−/−^ mice [12]. CDH11 might promote the secretion of interleukin- (IL-) 6, a critical inflammatory cytokine in the pathogenesis of RA [2]. Alternatively, CDH11 may induce the expression of IL-8, macrophage migration inhibitory factor (MIF) and monocyte chemotactic protein- (MCP-) 1 [3]. IL-8 and MCP-1 are chemokines that contribute to macrophage migration, an event that plays an important role in arthritis. Furthermore, IL-8 could promote the aggregation of neutrophils and angiogenesis [13].* MIF* ^−/−^ mice are protected from inflammation observed in the CIA model [14], indicating that MIF is involved in cell migration, infiltration, and inflammation processes. CDH11 induces a robust MIF expression, an effect that was not mediated by tumor necrosis factor- (TNF-) *α* [15].* CDH11* inactivation in mice prevents infiltration of inflammatory cells and erosion of cartilage by the pannus that are observed in the context of CIA mice [12]. These findings demonstrate that CDH11 is critical for the development of joint inflammation in RA. CDH11 could favor migration of FLS, thereby promoting the ability of these cells to erode cartilage [4]. CDH11 is expressed by multiple tumor cells of epithelial origin and correlated with poor differentiation and cancer aggressiveness [16]. This evidence illustrated that CDH11 is correlated to bone erosion and contributes to joint damage of RA. In addition, CDH11 activates MAPK and NF-*κ*B in FLS, thereby promoting secretion of matrix metalloproteinases (MMP) [17]. OA is a disease of joint degeneration characterized by joint inflammation and cartilage degradation, although these effects are milder than in RA. In vitro studies reported that levels of secreted proinflammatory cytokines and MMP are higher in FLS from RA than in those from OA patients [18]. In our study, we demonstrated higher levels of CDH11 in RA FLS both in vitro and in vivo. This event possibly leads to a positive feedback loop where FLS activation by CDH11 increases expression of inflammatory factors and MMP, thereby exacerbating inflammation and cartilage degradation in RA.

UCMSC are characterized by their self-renewal and multilineage differentiation potential, immunoregulatory properties, and low immunogenicity, making them promising candidates in therapeutic approaches in the field of cell therapy and tissue engineering [19]. Previous researches showed that UCMSC ameliorated symptoms of systemic lupus erythematosus (SLE) [20] and Crohn's disease [21] by secreting soluble factors that mitigate lymphocytes dysfunction. IL-10 secreted by Th2 cytokine inhibits Th1 cells and the activity of inflammatory cytokines, such as interferon-*γ*, IL-2, IL-12, and TNF-*α* [22].* IL-10* ^−/−^ mice are sensitized to the joint inflammation caused by CIA. Accordingly, inhibition of IL-10 expression in the synovium induces IL-1 and TNF-*α* levels, two major dominant proinflammatory cytokines in RA [23], indicating that IL-10 has a protective function in RA. We report that CDH11 expressed by FLS from RA patients is downregulated following coculture with UCMSC, an effect mediated by soluble factors. IDO, HGF, and IL-10 are regulatory molecules secreted by UCMSC [24, 25], and we observed that levels of these factors were upregulated following coculture with FLS. Suppression of IL-10, but not of IDO or HGF, activity precluded the inhibitory function of UCMSC on CDH11 expression. Accordingly, synovium from rats subjected to CIA expressed higher levels of CDH11, an event observed in RA patients. UCMSC transplantation downregulated CDH11 expression levels in the synovium, suggesting that UCMSC inhibit CDH11 expression in RA FLS by secreting IL-10. This event precludes the ability of FLS from RA patients to migrate and erode cartilage, thereby improving arthritis.

## 5. Conclusion

IL-10 mediates the inhibitory effect of UCMSC on CDH11 expression by FLS from RA patients, and this mechanism might be targeted to ameliorate arthritis.

## Figures and Tables

**Table 1:** Primers for real-time PCR.

Gene	Forward	Reverse
h-GAPDH	GCACCGTCAAGGCTGAGAAC	TGGTGAAGACGCCAGTGGA
h-CDH11	GTGCATGCCAAAGACCCTGA	CTGCTGCAAAGACAGTGATGTTGA
IDO	GAATGGCACACGCTATGGAA	CAGACTCTATGAGATCAGGCAGATG
COX2	TGACCAGAGCAGGCAGATGAA	CCACAGCATCGATGTCACCATAG
HGF	GTCAGCCCTGGAGTTCCATGATA	AGCGTACCTCTGGATTGCTTGTG
IL-10	GAGATGCCTTCAGCAGAGTGAAGA	AGTTCACATGCGCCTTGATGTC
TGF-*β*	AGCGACTCGCCAGAGTGGTTA	GCAGTGTGTTATCCCTGCTGTCA
HLA-G	CCTTGCAGCTGTAGTCACTGGA	CACACAGGGCAGCTGTTTCA
r-GAPDH	GGCACAGTCAAGGCTGAGAATG	ATGGTGGTGAAGACGCCAGTA
r-CDH11	TGCTGCCAACAGCCCAATAA	GAGATGTTGAGCCAGGCAGTTTC

h: human; r: rat.
